# cAMP-specific phosphodiesterase 8A and 8B isoforms are differentially expressed in human testis and Leydig cell tumor

**DOI:** 10.3389/fendo.2022.1010924

**Published:** 2022-10-07

**Authors:** Federica Campolo, Chiara Capponi, Maria Grazia Tarsitano, Marta Tenuta, Carlotta Pozza, Daniele Gianfrilli, Fabio Magliocca, Mary A. Venneri, Elena Vicini, Andrea Lenzi, Andrea M. Isidori, Federica Barbagallo

**Affiliations:** ^1^Department of Experimental Medicine, Sapienza University of Rome, Rome, Italy; ^2^Department of Anatomical, Histological, Forensic and Orthopedic Sciences, Sapienza University of Rome, Rome, Italy; ^3^Department of Medical and Surgical Sciences, University Magna Graecia of Catanzaro, Catanzaro, Italy; ^4^Department of Radiological, Oncological and Pathological Sciences, Sapienza University of Rome, Rome, Italy; ^5^Faculty of Medicine and Surgery, Kore University of Enna, Enna, Italy

**Keywords:** PDE8A, PDE8B, human testis, acrosome biogenesis, Leydig cell, Leydig cell tumor

## Abstract

Cyclic adenosine monophosphate/Protein kinase A (cAMP/PKA) signaling pathway is the master regulator of endocrine tissue function. The level, compartmentalization and amplitude of cAMP response are finely regulated by phosphodiesterases (PDEs). PDE8 is responsible of cAMP hydrolysis and its expression has been characterized in all steroidogenic cell types in rodents including adrenal and Leydig cells in rodents however scarce data are currently available in humans. Here we demonstrate that human Leydig cells express both PDE8A and PDE8B isoforms. Interestingly, we found that the expression of PDE8B but not of PDE8A is increased in transformed Leydig cells (Leydig cell tumors-LCTs) compared to non-tumoral cells. Immunofluorescence analyses further reveals that PDE8A is also highly expressed in specific spermatogenic stages. While the protein is not detected in spermatogonia it accumulates nearby the forming acrosome, in the trans-Golgi apparatus of spermatocytes and spermatids and it follows the fate of this organelle in the later stages translocating to the caudal part of the cell. Taken together our findings suggest that 1) a specific pool(s) of cAMP is/are regulated by PDE8A during spermiogenesis pointing out a possible new role of this PDE8 isoform in key events governing the differentiation and maturation of human sperm and 2) PDE8B can be involved in Leydig cell transformation.

## Introduction

Mammalian spermatogenesis is a complex differentiation process that involves the interplay of different cell types and comprise a series of cellular and biochemical metamorphoses.

A long series of findings suggest that among the delicate regulatory mechanisms implicated in testicular function, the second messengers cyclic adenosine monophosphate (cAMP) and cyclic guanosine monophosphate (cGMP) dependent signal transduction pathways play a role of key importance (1–4). In the somatic cells, Leydig cells (LCs) and Sertoli cells, they promote steroidogenesis when Luteinizing hormone (LH), released by pituitary gland, binds its receptor (LHR). The subsequent activation of adenylate cyclase (ACs) leads to an increase of cAMP level, that in turn allows protein kinases (PKA) to phosphorylate its target substrates (5). In the seminiferous tubules, cAMP and cGMP cooperate in the control of germ cell differentiation, both directly and indirectly through Sertoli cells (6). Sharper evidences have been reported in their involvement in sperm function during capacitation, such as activation of motility, changes in the motility pattern known as hyperactivation and for development of the ability to undergo the acrosome reaction (7).

The fine local and temporal regulation of cAMP/cGMP depends on the net balance between their generation by cyclase enzymes, Adenylate cyclases-ACs and Guanylate cyclases-GCs, and hydrolysis by the phosphodiesterases (PDEs) (5). PDEs are a class of enzymes belonging to 11 different gene families (PDE1-11) that undergo several alternative splicing giving rise to multiple isoforms in human and rodents (8–10) displaying, in some cases, specific cellular and subcellular distribution (11–14). The expression of these enzyme in human testis has been reported for a restricted group (PDE1, PDE4, PDE5 and PDE11) and their role was attributed in controlling sperm function for (15–17).

Although the role of some PDEs in rodent testis has been already elucidated, data on humans are scarce. Starting from the above findings, in this study, we investigated the expression of PDEs in human testis using different approaches.

## Materials and methods

### Ethical approval

The use of testicular tissues for this study was approved by a regional medical and research ethics committee (permit no. H-2-2014-103) and by the Policlinico Umberto I Ethical Committee. Normal testicular biopsies were obtained from heart-beating organ donors at the hospital Policlinico Umberto I. Biopsies were fixed in 4% paraformaldehyde (PFA) (Electron Microscopy Sciences, Hatfield, PA, USA)) and paraffin-embedded. All the samples showed well-preserved testicular tissue and a normal spermatogenesis. Residual tissues from orchiectomy specimens of patients diagnosed with Leydig cell tumours (LCT) were collected after their written informed consent. After evaluation by pathologists, the remaining tissue fragments were snap-frozen for molecular analyses or fixed and paraffin-embedded for histological examinations.

### QuantiGene 2.0 plex assay

Expression of Phosphodiesterases on human testis (n=4) or Leydig cells tumours (n=4) was analyzed by a custom QuantiGene Plex Assay (QGPA,Affymetrix, Santa Clara, CA, USA) according to manufacturer’s instructions. 5 mg human testis biopsies from Leydig cell tumours and healthy donors were processed. The assay was performed in a 96-well plate including three replicates for each sample, signal was detected by a Bio-Plex 200 SystemTM (BioRad, Hercules, CA, USA.) and data analysis was performed using the Bio-Plex ManagerTM 6.0 software (BioRad, Hercules, CA, USA). Results were normalized for HPRT1 expression.

### Real time PCR

Total RNA was isolated from human testis biopsies (NT, n=9; LCTs n=9) using Rneasy isolation kit (Qiagen, Hilden, Germany) according to manufacturer’s instruction. RNA was treated with DNAse (Zymo Research, CA, USA) and reverse transcribed with random hexamer primers using GoScript I Reverse Transcriptase (Promega, Mannheim, Germany). After cDNA synthesis step, qRT-PCR reaction was carried out in triplicate for each gene for each sample by using GoTaq qPCR Master Mix (Promega, Mannheim, Germany) using HPRT1 for normalization. The PCR reaction was carried out using the CFX Connect Real Time PCR System (BioRad, Hercules, CA, USA). Primers pairs used for qRT-PCR are: hPDE8ART-Fw 5’-TTGTTGGTGTAGTACGCAGG-3’, hPDE8ART-Rw 5’-CAGCTGGAGCAGTTCATTGT-3’; hPDE8BRT-Fw 5’-CAACAGCACGTGAAGATCAC-3’,hPDE8BRT-Rw 5’-CAATGGACTCTTTCCTCCTG-3’; hHPRT1RT-Fw 5’-GTCTTGCTCGAGATGTGATG-3’, hHPRT1 RT Rw 5’-GTAATCCAGCAGGTCAGCAA. For quantification analysis, the comparative threshold cycle (Ct) method was used. The Ct values of each gene were normalized to the Ct value of HPRT1. The gene expression levels were evaluated by the fold change using the equation 2^−ddCt^.

### Immunofluorescence analysis

Immunofluorescence on human testis sections (n=4) was performed after deparaffinization and rehydration by decreasing alcohol grades. Antigen retrieval was performed microwaving sections immersed in 10mM Sodium Citrate Buffer (pH 6.0). Permeabilization was performed with 0.1% Triton v/v for 10 minutes and blocking was achieved with 5% Donkey Serum-PBS v/v solution for 30 minutes (Sigma Aldrich, MI, USA). Sections were incubated with primary antibodies overnight at 4°C: anti-PDE8A (Atlas Antibodies Cat#HPA007722, RRID : AB_1855130), PDE8B (Atlas Antibodies Cat#HPA036912, RRID : AB_10670377), anti-ACROSIN (Biosonda, Santiago, Chile; Cat No. AMC-ACRO-C5F10-AS), Anti-Golgin-97 (SC-59820, Santa Cruz Biotechnology), anti-GM130 (610822 BD Biosciences), Lectin-PNA Alexa Fluor 488 (L21409 ThermoFisher Scientific). After overnight incubation at 4°C, sections were washed in PBS and incubated with Alexa Fluor 488-Donkey anti-Rabbit IgG, Alexa Fluor 568-Donkey anti-Mouse IgG or Alexa Fluor 568-Donkey anti-Goat IgG antibodies (Thermo Fisher Scientific, Waltham, MA, USA) for 1h at room temperature. Following extensive washes in PBS, sections were counterstained with DAPI and mounted with VECTASHIELD^®^ Antifade Mounting Medium (Vector Laboratories, Newark, CA, USA). Confocal images were acquired as z-stacks on Zeiss Airyscan 2 with a 40x immersion oil objective (Carl Zeiss, Oberkochen, Germany). The acquisition was performed for 10 z-stack with a z-step of 0.5 mm and a frame size of 1024x1024 pixel. Pachytene spermatocytes and spermatids were identified by nuclear morphology as previously described (18, 19).

### Immunohistochemistry analysis

Human testis biopsies from LCTs patients (n=9) and healthy donors (n=4) after fixation were embedded in paraffin (Bio Optica, Milano, Italy). Sections, obtained with the HM355S Microtome (Thermo Fisher Scientific, Waltham, MA, USA), were de-waxed, re-hydrated and finally processed for IHC using the EnVision^®^+ Dual Link System-HRP (DAB+) (DAKO/Agilent, Santa Clara, CA, USA) according to manufacturer’s instructions. After each step sections were washed three times with (PBS) 0,05% v/v Tween20. Antigen retrieval was performed by microwaving sections in 10mM Sodium Citrate pH 6.0 0,05% Tween20 v/v for 10 minutes. Sections were incubated overnight at 4°C with primary antibodies. Antibodies dilutions were performed in Bond Primary Antibody Diluent (Leica, Wetzlar, Germany). Before mounting, slides were counterstained with Hematoxylin (Sigma Aldrich, Saint Louis, MO, USA). Images were acquired by Zeiss Axiovert 200 inverted microscope using ZEN imaging software (Carl Zeiss., Oberkochen, Germany).

### Statistical analyses

Data are expressed as median and 5-95 percentile, or mean ± SEM as appropriate. Distribution of data was assessed by Shapiro-Wilk test. Differences in outcomes of interest were analyzed by Mann-Whitney test according to the distribution of data. Differences were considered significant with * p<0.05.

## Results

### Phosphodiesterases expression in human testis

**Figure 1:**
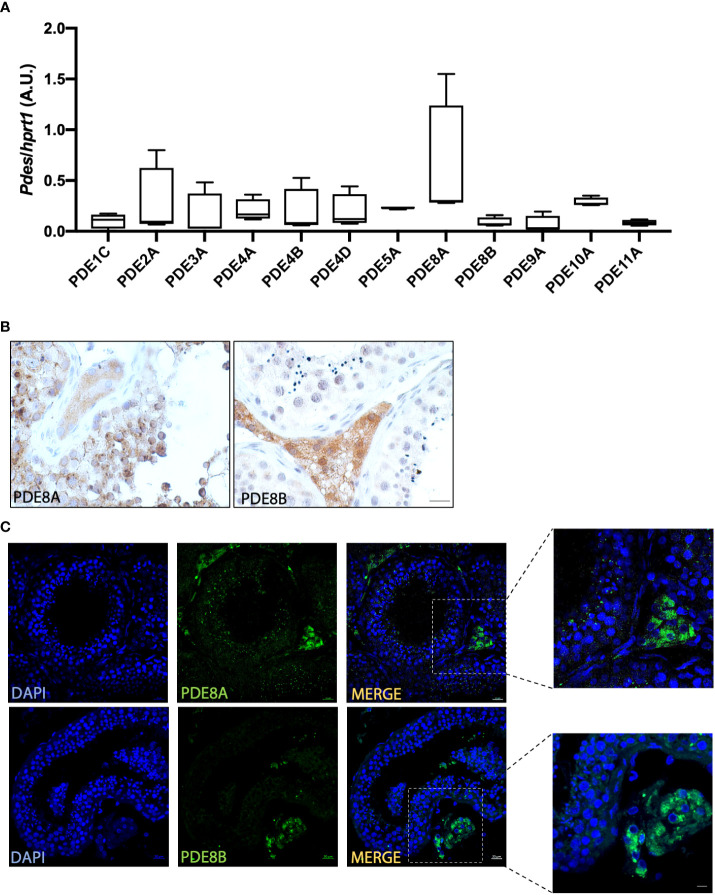
PDEs expression analysis in human testis **(A)** PDEs mRNA expression analysis by QuantiGene Bioplex Assay on human testis biopsies from healthy subjects. Results, normalized for HPRT1, are expressed as arbitrary units (A.U, (median and 5-95 percentile) and reported in the histogram bar chart (n=4). **(B)** PDE8 isoforms expression analysis by immunohistochemistry using commercially available anti-PDE8A and anti-PDE8B antibodies on human testis biopsies. Scale bars represent 20 μm. **(C)** Localization of PDE8 isoforms by immunofluorescence analysis on sections obtained from human testis biopsies of healthy subjects. PDE8 isoforms staining is shown in green and nuclei are shown in blue (DAPI). Scale bars represent 20 μm except for the insets where they represent 10 μm.

PDEs transcripts have been previously detected in mouse and rat testis but their expression in human testis has not been investigated yet. To overcome this gap in literature we firstly analyze PDEs expression by a QuantiGene Plex Assay (QGPA) on healthy donor biopsies (**Figure 1A**). This initial characterization revealed that all PDEs transcripts analyzed were detected by QGPA in normal testis samples, even if their expression level varies profoundly according to the isoforms (**Figure 1A**). As previously reported for other species PDE8A was detected at high level also in human testicular lysates and the other PDE8 isoform, PDE8B, was slightly detectable suggesting that the distribution of this isoform was restricted to specific cell types. Thus, further analyses of expression pattern were conducted for PDE8A and PDE8B isoforms.

### PDE8 isoforms are differentially expressed in human testis

To deeply investigate PDE8 expression pattern, immunohistochemistry using specific antibodies for PDE8A and PDE8B was performed on human testis sections. This analysis revealed that PDE8A signal was localized within the seminiferous tubules and in LCs, while PDE8B was exclusively detected in LCs in sections derived from healthy subject (**Figure 1B**). Analogous results were obtained by immunofluorescence experiments. As shown in **Figure 1C**, PDE8A immunoreactivity was not restricted to interstitial cells, as previously reported for mouse testis, but a specific staining was also detected in germ cells, specifically in pachytene spermatocytes and in spermatids. Higher magnification confirmed the expression pattern of PDE8A- enriched in perinuclear granules of germ cells and diffused in the cytoplasm of LCs- and of PDE8B whose staining was detected only in the cytosolic region of LCs (**Figure 1C**, inset).

**Figure 2:**
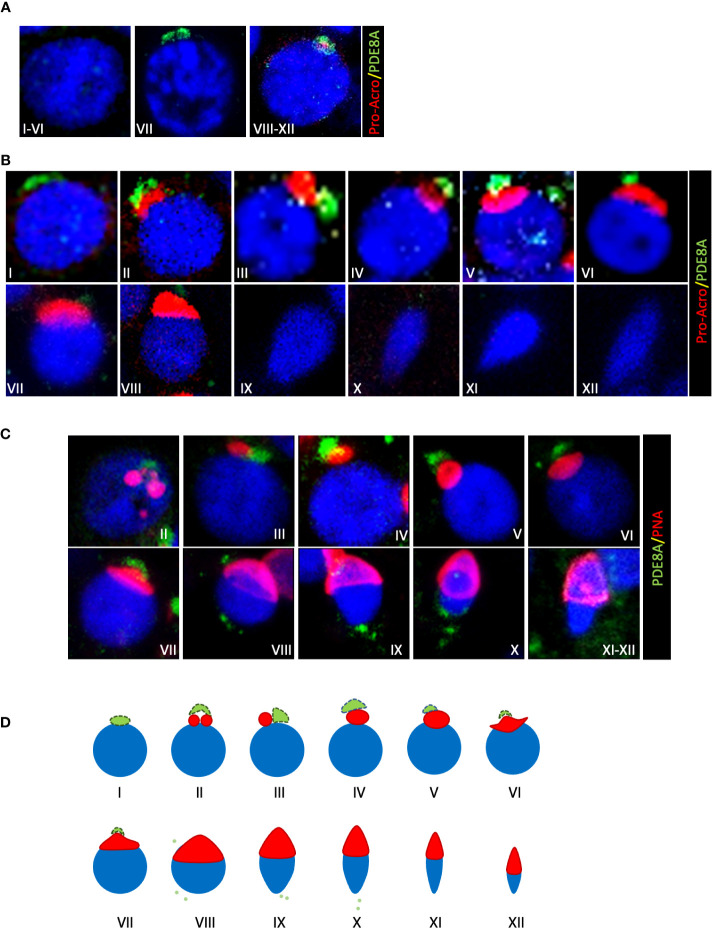
Localization of PDE8A in different stages of human spermatogenesis **(A)** PDE8A localization in human spermatocytes, to better show the differences between them the stages of the whole spermatogenic cycle are arbitrarily sub-divided into three parts. Merged fluorescent channels DAPI-nuclear staining (blue), pro-acrosin staining (red) and PDE8A fluorescence (green) are shown. **(B)** PDE8A localization in human spermatids during the whole spermatogenic cycle are classified according to pro-acrosin staining. Merged fluorescent channels DAPI-nuclear staining (blue), pro-acrosin staining (red) and PDE8A fluorescence (green) are shown. **(C)** PDE8A localization in human spermatids during the whole spermatogenic cycle classified according to PNA staining. Merged fluorescent channels DAPI nuclear staining (blue), PNA staining (red) and PDE8A fluorescence (green) are shown. **(D)** PDE8A localization (green/light green) in spermatids in comparison with a schematic representation of the different phases of acrosome biogenesis (red).

Based on pro-acrosin staining (18) all stages of seminiferous tubules were then analyzed for PDE8A localization. Confocal images revealed a positive staining in pachytene spermatocytes from stage VII to stage XII. At stage VII, when pro-acrosin is not yet detectable in pachytene spermatocytes, PDE8A forms a two beads perinuclear structure. From stage VIII a spherical PDE8A case embeds the punctate pattern of pro-acrosin that last until stage XII (**Figure 2A**). Later, PDE8A expression in perinuclear granules gradually increases in round spermatids from stage I to V and decreases thereafter (**Figure 2B**). It is worth to note that a highly dynamic structure was found for PDE8A staining. Indeed, PDE8A antibody labeles a ribbon shape structure in early round spermatids (stage I) located perinuclearly. This structure remains in proximity with the forming acrosome vesicle later on, but it starts to move apart from the perinuclear region from stage II up to stage VIII concentrating above the acrosome. Pro-acrosin staining facilitates the recognition of spermatids until stage VIII but not in later steps of spermatids development (12). To better understand the fate of PDE8A in later stages of spermatid development, we performed co-staining analysis with Peanut agglutinin (PNA) lectin dye, which recognizes galactose, that binds to intra-acrosomal glycoprotein, allowing the visualization of the acrosome in elongating spermatids (20). PNA staining revealed that in steps IX-X spermatids, PDE8A immunoreactivity translocate in the caudal part of the cell, locating on the opposite side of the formed acrosome (**Figure 2C**). This redistribution of PDE8A immunoreactivity parallels the translocation of the Golgi apparatus in the caudal region of elongating spermatids (21). The relative morphological changes of acrosome and PDE8A immunoreactivity are schematically represented in **Figure 2D**.

### PDE8A colocalize with the Trans-golgi apparatus marker Golgin-97

In order to determine the nature of the structure labeled with PDE8A antibody we decide to perform immunofluorescence co-staining with known marker of intracellular organelles.

**Figure 3:**
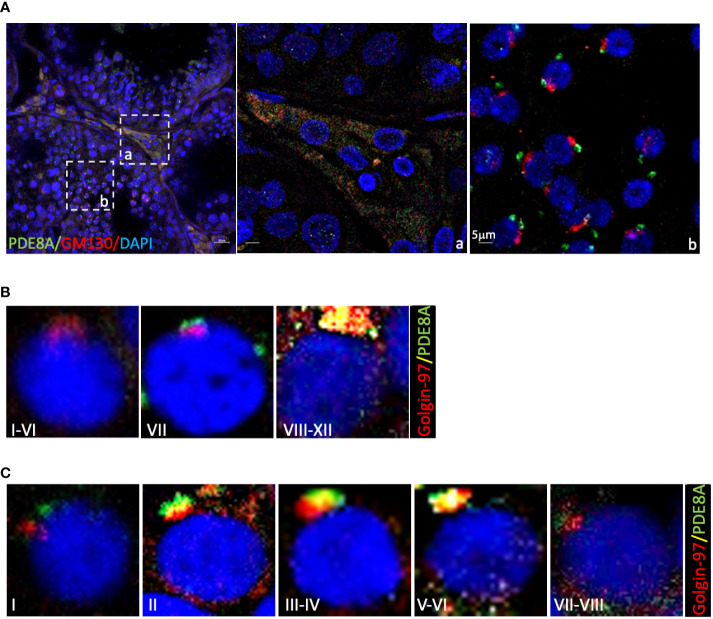
Co-localization of PDE8A and Golgi apparatus marker in different stages of human spermatogenesis **(A)** Co-localization analysis of PDE8A and GM130. Merged fluorescent channel DAPI-nuclear staining (blue), PDE8A (green) and GM130 staining (red) are shown. Scale bars represent 20 μm, except for the inlet **(A, B)**. **(B)** PDE8A and Golgin97 co-localization analysis in human spermatocytes, to better show the differences between them the stages of the whole spermatogenic cycle are arbitrarily sub-divided into three parts. Merged fluorescent channels DAPI-nuclear staining (blue), Golgin-97 (red) and PDE8A (green) are shown. **(C)** PDE8A and Golgin-97 co-localization analysis in human spermatids; to better show the differences between them the whole spermatogenic cycle are arbitrary sub-divided in five. Merged fluorescent channels DAPI-nuclear staining (blue), Golgin-97 staining (red) and PDE8A fluorescence (green) are shown.

Giving the similarity of PDE8A structure with Golgi apparatus, two Golgi probes were used: Golgi Matrix Protein of 130 kDa (GM130), labeling cis-Golgi, and Golgin-97 labeling the trans-Golgi apparatus (22). As expected GM130 stains a compact peri-nuclear structure in round spermatids but do not co-localize with PDE8A in none of the analyzed stages (**Figure 3A**, inlet b). On the contrary, PDE8A (and also PDE8B, **Figure S1**, inlet a) co-localizes with the Golgi apparatus in LCs, as appreciable from co-staining analysis with GM130 (**Figure 3A**, inlet a).

In spermatocytes, from stage I to VI, Golgin-97 labels a crescent-like structure that becomes a dense rounded structure in the following stages. From stage VIII to XII PDE8A and Golgin-97 labels the same structure indicating that both proteins belong to trans-Golgi apparatus (**Figure 3B**). In round spermatids the co-localization of these two proteins is null at stage I, begin to increase from stage II, reaching a perfect overlapping at stage V (**Figure 3C**). During the subsequent steps of spermatids development, the spermatids become faintly labeled with both by PDE8A and Golgin-97 antibodies, due to the fragmentation of the structure in smaller vesicles that start to migrate in the caudal part of the elongating spermatids.

### Clinical characteristics of LCTs

Leydig cell tumours (LCTs) represent the most common non-germ cell testicular tumors accounting for 3–22% of all testicular neoplasms (23–26). In recent years, a progressive rise in the diagnosis of LCTs has been observed. One possible explanation is the growing use of testis ultrasonography in the screening for various andrological disorders (27, 28). However, exposure to endocrine disruptors exposure has been claimed for the impairment of the Leydig cell compartments (29, 30).

Given the finding of PDE8A and PDEB expression in Leydig cells and their role in steroidogenesis we wonder if their expression was modified in LCTs where hormone production is impaired.

Clinical characteristics of each patient are listed in **Table 1**. Hormonal data revealed a trend toward increased FSH levels, suggesting an initial tubular defect. In all LCT patients’ serum tumour markers (β-human chorionic gonadotropin - β-HCG, placental alkaline phosphatase - PLAP, alpha-fetoprotein - AFP, carcinoembryonic antigen - CA, ferritin, and lactate dehydrogenase - LDH) were negative. LCTs were histologically confirmed by pathology analysis (not shown), according to Kim’s criteria (31). Immunochemistry for inhibin and calretinin were used as confirmatory markers for the diagnosis of LCTs. Mean size of the LCTs analyzed was 1.0±0.4 (cm±SD).

**Table 1:** Clinical features of LCTs cohort.

ID Patient	Race	Age (years)	FSH (mUI/ml)r.r 1.38-9.58	LH (mUI/ml)r.r 1.8-8.16	Total Testosterone (nmol/l)r.r 10.4-38.2
#1	Caucasian	24	18.6	3.3	16.7
#2	Caucasian	64	7.4	5.8	9.9
#3	Caucasian	49	4.4	5.1	22.1
#4	Caucasian	18	2.2	2.9	15.1
#5	Caucasian	40	16.9	3.5	15.3
#6	Caucasian	59	16.1	5.7	10.1
#7	Caucasian	61	15.6	4.1	19.3
#8	Caucasian	21	1.5	2.3	18.2
#9	Caucasian	29	4.4	3.9	17.9

FSH, follicle stimulating hormone; LH, luteinizing hormone; r.r, reference range.

### PDE8A and PDE8B expression in LCTs

**Figure 4:**
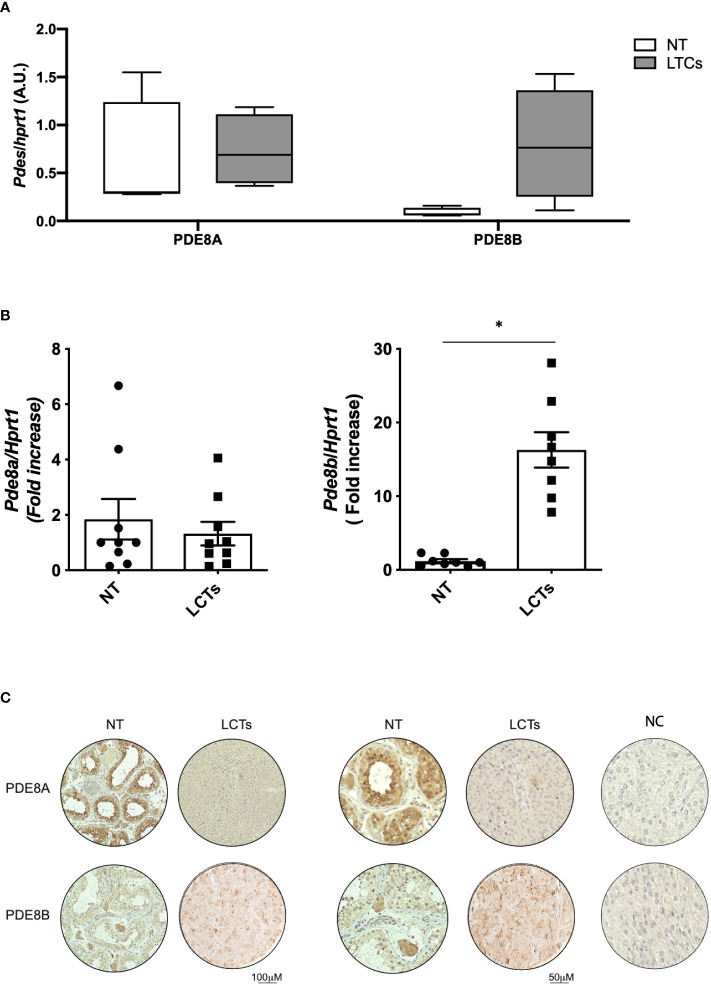
PDE8 expression analysis in LCTs **(A)** PDE8A and PDE8B mRNA expression analysis by QuantiGene Bioplex Assay on human testis biopsies from healthy subjects (NT) and LCT patients. Results, normalized for HPRT1, are expressed as arbitrary units (A.U, (median and 5-95 percentile) and reported in the histogram bar chart (n=4). **(B)** PDE8 isoforms mRNA expression analysis by qPCR, normalized for HPRT1, on human testis biopsies from healthy subjects (NT) and LCT patients. Results are reported as fold increase vs NT (mean ± SEM, n=9) in the histogram bar chart. *p<0.05. **(C)** PDE8 isoforms expression analysis by immunohistochemistry using commercially available anti-PDE8A and PDE8B antibodies on normal testis biopses (NT) and LCTs. Negative control (NC) is represented in the right panels. Scale bar are depicted beneath the panels.

QGPA assay on LCT biopsies using, as reference tissue, human testis biopsies from healthy subjects revealed that PDE8A expression was comparable to non-tumoral (NT) tissue. On the other hand, a marked increase of PDE8B was observed in LCTs compared to healthy testis (NT) (**Figure 4A**). To confirm this finding, RNA extracts were assayed for PDE8 isoforms by qPCR. According to the results obtained with QGPA, we found that PDE8B mRNA expression level was increased by 10-fold in LCTs compared to normal testis, while PDE8A levels were almost comparable between the two sample groups (NT vs LCTS) (**Figure 4B**).

Histological evaluation and immunohistochemistry analysis was performed on available LCTs sections (**Figure 4C** and **Figure S2**). When PDE8A and PDE8B staining was applied on LCTs samples, a more intense signal was appreciable for PDE8B isoform compared to non-tumoral tissue (**Figure 4C**) indicating that the up-regulation of the transcript is accompanied by a protein increase in dysfunctional tissue.

## Discussion

Several, if not all, PDEs have been shown to be express in rodent testis and their expression patterns and enzymatic characteristics have been shown to differ each other (32–38).

Substantial evidence for the regulatory function of PDEs in Leydig cells has been reported as a stimulatory effect of a pan-PDE inhibitor on testosterone release by primary LCs (39), indicating that one or more PDEs might be active in Leydig cells to modulate the intensity, duration and the desensitization of the LH-stimulated hormonal response (39). Indeed later on, it was demonstrated that Leydig cells express transcripts for several cAMP-specific PDEs such as Pde4a, Pde4b, Pde4d, Pde7a, Pde7b, Pde8a, and Pde8b, most of them contributing to Leydig cell response through LHR-cAMP signaling (40, 41). Among the cyclic guanosine monophosphate (cGMP) specific PDEs, PDE5A expression was found in Leydig and myoid cells of prepuberal and adult rat testis (38, 42). Both mRNA and protein levels of PDE5 significantly increased in isolated Leydig cells after testosterone treatment and were normalized by androgen receptor blockade, suggesting that testosterone may downregulate steroid synthesis by reducing cGMP production. The increased levels of PDE9 in Leydig cells isolated from testosterone-treated rats also confirm an active role of other cGMP-specific PDEs in degrading nitric oxide (NO)-stimulated cGMP (42). Regarding the role in germ cells, using specific inhibitors, have been suggested that PDE4 is involved in sperm motility, PDE1 would be involved in capacitation-associated modifications that occur in the acrosomal/head region of spermatozoa, PDE11 in sperm activation and PDE5 in motility, capacitation but not in acrosome reaction. Although PDE3 is detected in the postacrosomal region of the human sperm head, no specific role for this PDE in sperm function has been proposed so far (16, 17).

Scarce information regarding the presence, specific function and subcellular location of the PDE subtypes in human testis are available. In the present study we determined hPDEs expression in testis lysates using the QuantiGene Plex 2.0 assay (43, 44). This technique combining branched DNA (bDNA) signal amplification and multi-analyte profiling beads technologies enable the detection and quantitation of multiple mRNA targets simultaneously and the results obtained are more reliable since it reduces signal variability due to sample preparation, sample input inconsistency and/or overall, well/plate/experimental effects. The analyzed data confirmed that all PDEs transcripts tested were expressed, at different level, in human testis endorsing their fundamental role in testicular functions. Since, the highest expression level was observed for PDE8A we sought to better characterize PDE8 family expression and localization.

PDE8A and PDE8B, are transcribed from two different genes, which encode for two enzymes responsible for the highest affinity with the substrate among the cAMP-specific PDEs (45) and they play an important role in most of steroidogenic tissue (41, 46, 47). Northern blot analysis with a PDE8A probe on mRNA isolated from different murine tissues showed it is highly expressed in testis followed by liver, kidney, skeletal muscle, heart, eye, ovary, lung and brain, in descending order (48). In human, the same analysis showed the higher abundance in testis, ovary, colon and small intestine (49) indicating a similar but not totally overlapping expression pattern between human and mouse tissues. These differences can be attributable to the 80% sequence homology of PDE8A between the two species. For example, hPDE8A contains a REC domain that is not present in mPDE8A, nor in other PDEs, suggesting a unique regulation mechanism; on the other hand, mouse PDE8A protein, unlike the human enzyme, contains a nuclear localization consensus sequence (50). Soderling and co-workers described for the first time the presence of PDE8 transcripts in mouse testis revealing a stage-specific expression of this enzymes by *in situ* hybridizations in spermatocytes (48); only several years later PDE8A or PDE8B proteins was demonstrated to be expressed in murine Leydig cells (51). The authors taking advantage of the PDE8-selective inhibitor PF-04957325 and PDE8 knockout mice, revealed that the inhibition/deletion of PDE8 is accompanied by an increase in steroids production in in these cells. They also found that PDE8A is largely associated with mitochondria, whereas PDE8B is broadly distributed in the cytosol and for this reason they are able to control different cAMP pools cooperating to regulate steroids production (51). They also suggested that the use of PDE8 specific inhibitor could represent a functional strategy to restore normal steroidogenesis in human dysfunctional LCs.

In the present study, our immunohistochemistry results on human testis section clearly show that PDE8A and PDE8B are localized in the cytosol in granular structure in Leydig cells recapitulating what was already observed in mouse testis proposing an analogous role of these enzymes in steroidogenesis in the same fashion as in murine testis. Expression, localization and functional study on primary human cryopreserved Leydig cells would be helpful to confirm this hypothesis. PDEs overexpression has been already described in several cancer types (52–61); and their pharmacological inhibition has been shown to affect progression, migration, angiogenesis and differentiation in a large spectrum of tumor cells suggesting a potential application of PDE inhibitors as anticancer agents (62). Despite the fundamental role of cAMP and cGMP in regulating steroidogenesis and cell transformation, to the best of our knowledge, PDE8 role in LCs dysfunction was not evaluated so far. By comparing PDE8 isoform expression in human testis and LCTs, we have demonstrated by different approaches that PDE8B, but not PDE8A, is selectively increased in dysfunctional LCs. Since both isoforms are equally expressed in normal LCs, we are confident that the up-regulation of PDE8B is not due to an increased abundance of Leydig cells in LCTs compared to healthy tissue, but it is specifically due to a miss regulation of this enzyme in tumoral tissue. While PDE8B is increased in these sample we cannot ascertain if this result parallel with an increase of its activity. Owing to the difficulty in collecting LCTs tissue, the sample size was limited and the pathological role of PDE8B and its involvement in Leydig cells transformation need to be extended in future studies.

One of the most peculiar events occurring during spermatogenesis is spermiogenesis, the functional and structural changes that from round spermatids generate, elongated spermatids leading to spermatozoa production. A key event of this transformation is the acrosome biogenesis that starts in pachytene spermatocytes with the fusion of proacrosomal granules (21). During the “Golgi phase” the acrosomal vesicle tightly attaches to one pole of the nuclear envelope and grows thanks to the continuous arrival and fusion of new Golgi-derived vesicles; during the later stage “the cap phase” the granule initially enlarges becoming a flat cap that covers two-thirds of the nuclear envelope. During the maturation phase the acrosome spreads over the entire nuclear membrane while Golgi apparatus are eliminated as cytoplasmic droplets prior to spermiation in a region that is opposed to the formed acrosome. In this complex scenario become clear that Golgi apparatus and its associate protein hold a fundamental role of good-quality gametes production able to effectively fertilize the egg. Our immunofluorescence analyses reveal that PDE8A is expressed in round spermatids in the perinuclear region, close to acrosome but not coexisting with it, and associates with trans-Golgian region in specific stages (as revealed by Golgin-97 co-staining). In this context, it may support and sustain the trafficking of the vesicles originating from the Golgi apparatus for the acrosome biogenesis. Indeed, cAMP/PKA signaling pathway has been recognized as an outstanding member of signaling molecules in regulating Golgi stability and biogenesis. Its role in modulating the budding of transport vesicle has been elegantly demonstrated in several cell types (63). Additionally, using PF-04957325 on LCs, several modification of phosphorylation status of regulators of Golgi assembly/reassembly were identified (64), reinforcing the hypothesis that PDE8A can modulate these processes. Human PDE8 enzyme has high hydrophilicity and surface probability existence and its possible membrane localization is further supported by several potential sites for myristylation and palmitoylation (50) that can favor the interaction with Golgi membranes.

A vast array of specific proteins is involved in acrosome biogenesis and any defect may result in malformation of the acrosome and eventually lead to infertility. For example, has been demonstrated that deficient mice for Gopc (Golgi-associated PDZ- and coiled-coil motif-containing protein) that is a Golgi-associated protein and for Agfg1, that is required for docking and/or fusion of pro-acrosomic vesicles during acrosome biogenesis, are infertile due to globozoospermia (65). It would be interesting to identify mutation on PDE8A gene related to patients affected by globozoospermia or other infertility defects.

This is the first study analyzing the expression of PDEs in normal and tumoral human testis. Using different experimental approaches, our findings suggest that 1) a specific pool(s) of cAMP is/are regulated by PDE8A during spermiogenesis pointing out a possible new role of this PDE8 isoform in key events governing the differentiation and maturation of human sperm and 2) PDE8B can be involved in Leydig cell transformation.

## Data availability statement

The original contributions presented in the study are included in the article/**Supplementary Material**. Further inquiries can be directed to the corresponding author.

## Ethics statement

The studies involving human participants were reviewed and approved by regional medical and research ethics committee Policlinico Umberto I Ethical Committee. The patients/participants provided their written informed consent to participate in this study.

## Author contributions

FC and CC, methodology and analysis, data curation, and draft editing. MGT, methodology and draft editing. MT, CP, and DG, patient recruitment. FM, investigation. MV, AL, EV, and AI, resources and draft editing. FB, conceptualization, analysis, data curation, draft writing and editing, funding acquisition. All authors contributed to manuscript revision, read, and approved the submitted version.

## Funding

This work was supported by research grants from Ministry of University and Research (FIRB2012 RBFR12FI27, PRIN2020 20203AMKTW to FB, PRIN2017 2017TK7Z8L to CP), Umberto Veronesi Foundation 2019 to FB, Bando Ateneo Sapienza University 2020 “Avvio alla Ricerca” to FC.

## Acknowledgments

We thank Susanna Dolci and Flavia Botti for helpful suggestions.

## Conflict of interest

The authors declare that the research was conducted in the absence of any commercial or financial relationships that could be construed as a potential conflict of interest.

## Publisher’s note

All claims expressed in this article are solely those of the authors and do not necessarily represent those of their affiliated organizations, or those of the publisher, the editors and the reviewers. Any product that may be evaluated in this article, or claim that may be made by its manufacturer, is not guaranteed or endorsed by the publisher.
