# Supplementary material for: cAMP-specific phosphodiesterase 8A and 8B isoforms are differentially expressed in human testis and Leydig cell tumor
Source: Front Endocrinol (Lausanne). 2022 Oct 7;13:1010924. doi: 10.3389/fendo.2022.1010924 (PMC9585345; doi:10.3389/fendo.2022.1010924)
Supplement: Supplementary file 1 [file DataSheet_1.pdf]

Supplementary Material

1.1 Supplementary Figures

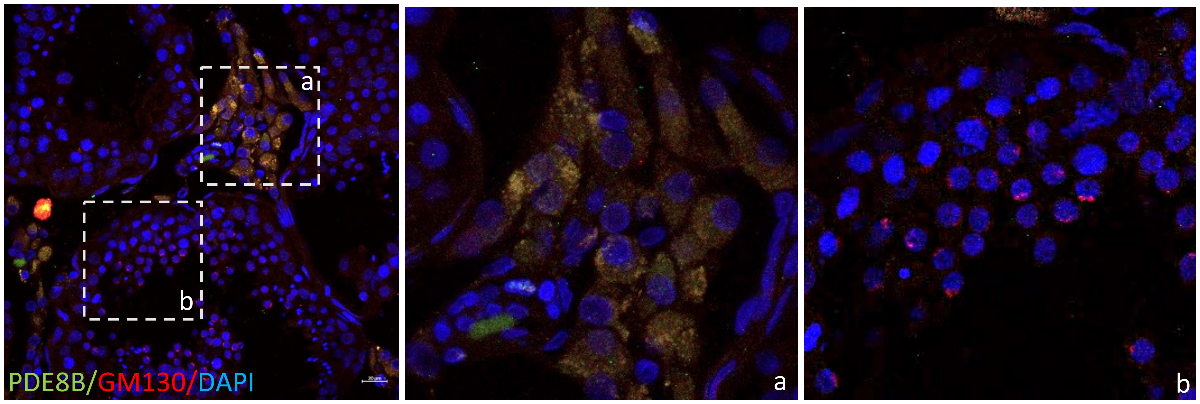

Supplementary Figure 1. Co-localization analysis of PDE8B and GM130. Merged fluorescent channels DAPI- nuclear staining (blue), GM130 staining (red) and PDE8B (green) are shown. Scale bars represent 20 µm, except for the inlet (a, b).

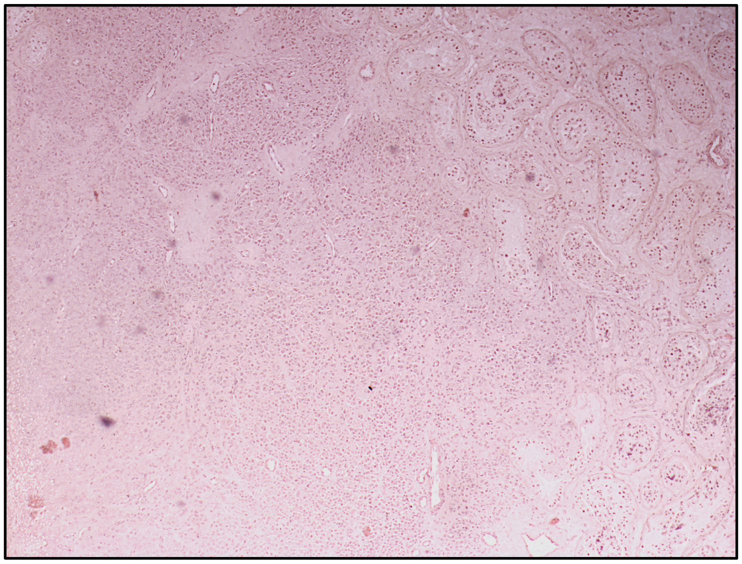

Supplementary Figure 2. Representative Hematoxylin and Eosin staining of LCTs. Magnification 4X
